# Reducing TDP-43 aggregation does not prevent its cytotoxicity

**DOI:** 10.1186/2051-5960-1-49

**Published:** 2013-08-09

**Authors:** Rui Liu, Guang Yang, Takashi Nonaka, Tetsuaki Arai, William Jia, Max S Cynader

**Affiliations:** 1 Brain Research Center, University of British Columbia, 2211 Wesbrook Mall, Vancouver, BC V6T2B5, Canada; 2 Department of Neuropathology and Cell Biology, Tokyo Metropolitan Institute of Medical Science, Setagaya-ku, Tokyo 156-8506, Japan; 3 Department of Psychiatry, Graduate School of Comprehensive Human Sciences, University of Tsukuba, 1-1-1 Tennoudai, Tsukuba, Ibaraki 305-8575, Japan

**Keywords:** TDP-43, Aggregation, Peptides, Cell death

## Abstract

**Background:**

TAR DNA-binding protein 43 (TDP-43) is a protein that is involved in the pathology of Amyotrophic Lateral Sclerosis (ALS) and Frontotemporal Lobar Degeneration (FTLD). In patients with these neurodegenerative diseases, TDP-43 does not remain in its normal nuclear location, but instead forms insoluble aggregates in both the nucleus and cytoplasm of affected neurons.

**Results:**

We used high density peptide array analysis to identify regions in TDP-43 that are bound by TDP-43 itself and designed candidate peptides that might be able to reduce TDP-43 aggregation. We found that two of the synthetic peptides identified with this approach could effectively inhibit the formation of TDP-43 protein aggregates in a concentration-dependent manner in HeLa cells in which a mutated human TDP-43 gene was overexpressed. However, despite reducing aggregation, these peptides did not reduce or prevent cell death. Similar results were observed in HeLa cells treated with arsenite. Again we found reduced aggregation, in this case of wild type TDP-43, but no difference in cell death.

**Conclusions:**

Our results suggest that TDP-43 aggregation is associated with the cell death process rather than being a direct cause.

## Background

Recent evidence links TDP-43 pathology to at least two forms of neurodegeneration that had heretofore been thought to be quite separate. FTLD is the second most common type of early-onset neurodegenerative dementia after Alzheimer’s disease, and ALS is the most common adult-onset progressive motor neuron disease (MND). The TAR DNA binding protein 43 (TDP-43) has been found to be the major protein constituent of the intracellular aggregated inclusions in both FTLD with ubiquitin-positive inclusions (FTLD-U) and ALS [1,2].

TDP-43 is a 414 amino acid protein encoded by the TARDBP gene on chromosome 1. It was originally identified as a transcriptional repressor of the human immunodeficiency virus type 1 (HIV-1) gene [3,4] and the mammalian gene SP-10 [5]. TDP-43 normally is found in the nucleus where it regulates RNA splicing, mRNA stability and microRNA processing [6-9], but TDP-43 in pathological inclusions is generally hyperphosphorylated, ubiquitinated [10], and cleaved to 35 and 25 kDa species [11]. The pathological aggregates are frequently found in the cytoplasm rather than at TDP-43’s normal nuclear location [1,2,12].

The mechanism through which TDP-43 is involved in neuronal death and degeneration remains unclear. One of the best characterized pathological features of TDP-43 proteinopathies is the cytoplasmic inclusions of TDP-43 aggregates. As with other protein misfolding diseases, TDP-43 mediated toxicity may result from a toxic gain of function associated with its aggregation. Johnson and colleagues have established a yeast model involving overexpressed full-length human TDP-43 or various TDP-43 truncation products [13]. They found that expressing a truncated form of TDP-43 containing both the C-terminal and RRM2 promoted aggregation. Only the aggregated form of TDP-43 induced toxicity to yeast cells. This suggested that TDP-43 misfolding and aggregation might be an important cause of cell death in neurodegenerative diseases. Another group supported this conclusion in human cell models [14]. The 25 kDa C-terminal fragment of TDP-43, which is likely to be a caspase-3 cleavage product [15], was overexpressed in HEK293 and differentiated M17 neuroblastoma cells. The 25 kDa fragment of TDP-43 formed cytoplasmic inclusions, induced cell toxicity, but did not disturb endogenous TDP-43 functions. Moreover, it was found that aggregation of mitochondria seems to be a common feature when overexpressing TDP-43 in transgenic mice [16,17]. All these data point to the toxicity of TDP-43 aggregates, or of the processes by which they form.

To further elucidate the links between TDP-43 aggregation and disease mechanisms, we used two different models. The first was an aggregation model pioneered by Nonaka and colleagues [18]. They found that a mutant TDP-43 lacking residues 187–192 formed intranuclear dot-like inclusions when expressed in SY5Y cells. A second model relied in recent findings that arsenite treatment can induce aggregation of wild-type TDP-43. When HeLa cells are treated with arsenite, an established stress granule (SG) inducer [19,20], TDP-43 aggregation occurs in the cytoplasm. Arsenite reacts with oxygen, induces oxidative stress and then activates HRI (Heme-regulated inhibitor), whose expression is required for SG formation [21]. SGs are cytoplasmic sites of stalled mRNA pre-initiation complexes induced by multiple stressors. Recent data have shown that SGs participate in the process of TDP-43 accumulation [22,23]. With these two models in hand, we asked whether selectively blocking aggregation would affect cellular toxicity. Inhibition of TDP-43 aggregation is considered to be a major potential therapeutic avenue for ALS and FTLD-U. Similar to the situation for other neurodegenerative diseases, potential tools include antibodies, molecular chaperones, chemical compounds and synthetic peptides. The strategy we used to design aggregation blockers was to identify and synthesize TDP-43 fragments that bind with full length TDP-43 protein. Our findings indicate that the synthetic peptides reduced the formation of TDP-43 aggregates in both models used, but did not reduce or prevent cell death.

## Results

### TDP-43 protein binds with selected TDP-43 derived peptides on the membrane

**Figure 1:**
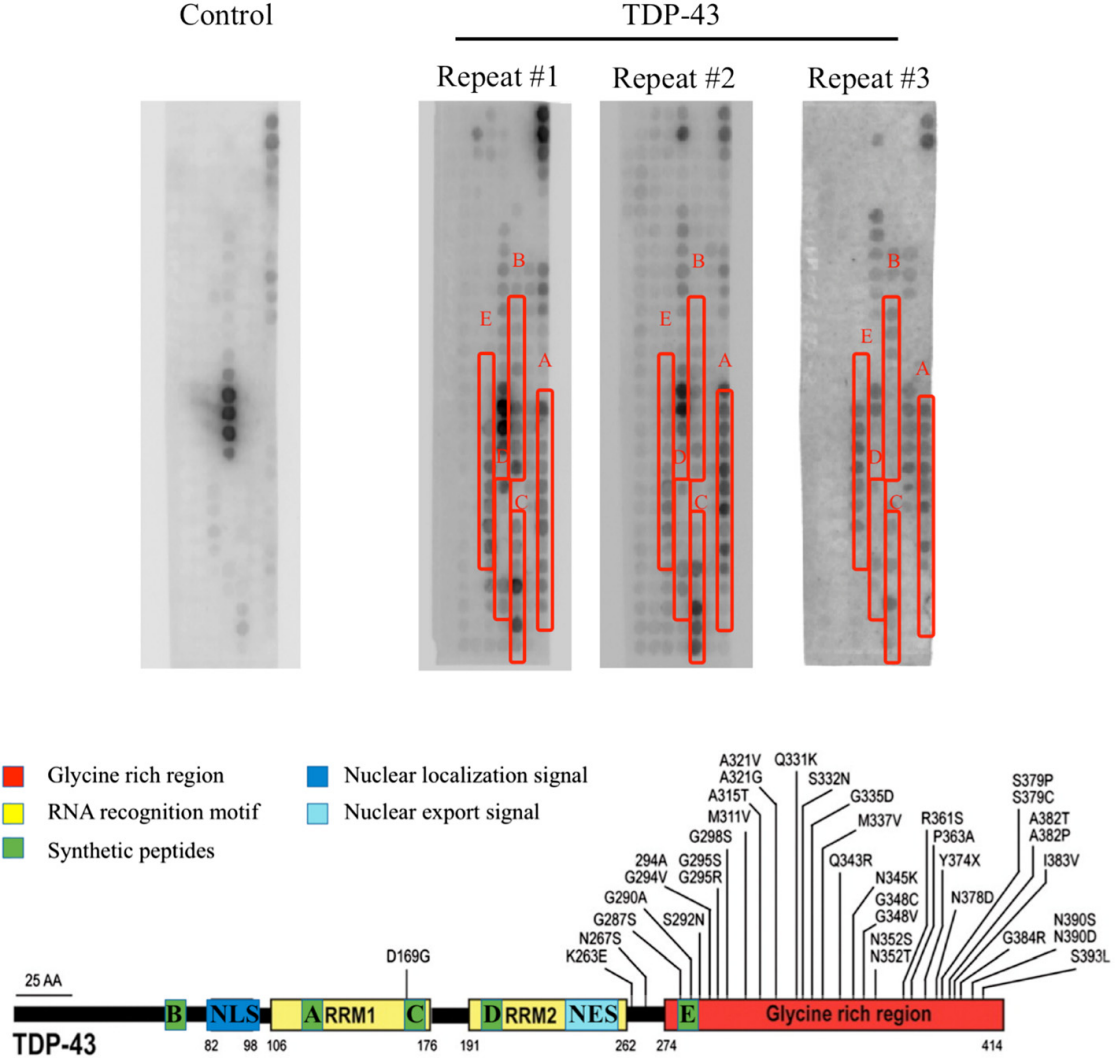
**Identification of peptide candidates that may block TDP-43 binding to itself using the high density peptide array.** TDP-43 protein associates with TDP-43 membrane at specific regions. Control, binding of the antibody only to the TDP-43 membrane; TDP-43, The TDP-43 membrane was incubated with TDP-43 protein (three repeats). The schematic representation shows the five peptide candidates labeled on the membranes. The peptides were designed based on the identified regions of the membrane to which purified TDP-43 bound.

The protein array described in the Methods section was used to identify candidate binding regions at which TDP-43 might interact with itself. Given the nature of the overlapping sequences associated with each neighboring peptide (frame shift of 2 aa), only positive spots in a series were considered as possible binding sites. Five separate and robust binding regions were found on the TDP-43 peptide array after incubating the membrane with recombinant TDP-43 protein followed by visualization of an antibody against TDP-43 (Figure 1). This result suggests that the five regions may potentially be involved in self binding or interaction of TDP-43. Based on these possible interaction domains, five peptide candidates, called Peptide A-E, were designed and synthesized. The peptides were derived from the sequence of full length TDP-43. The overlapping amino acids are the possible binding domains and are indicated in Figure 1. The distribution of five peptides is shown on the schematic of TDP-43 structure.

### The synthetic peptides were able to block the interaction between recombinant TDP-43 protein and its peptide array membrane

**Figure 2:**
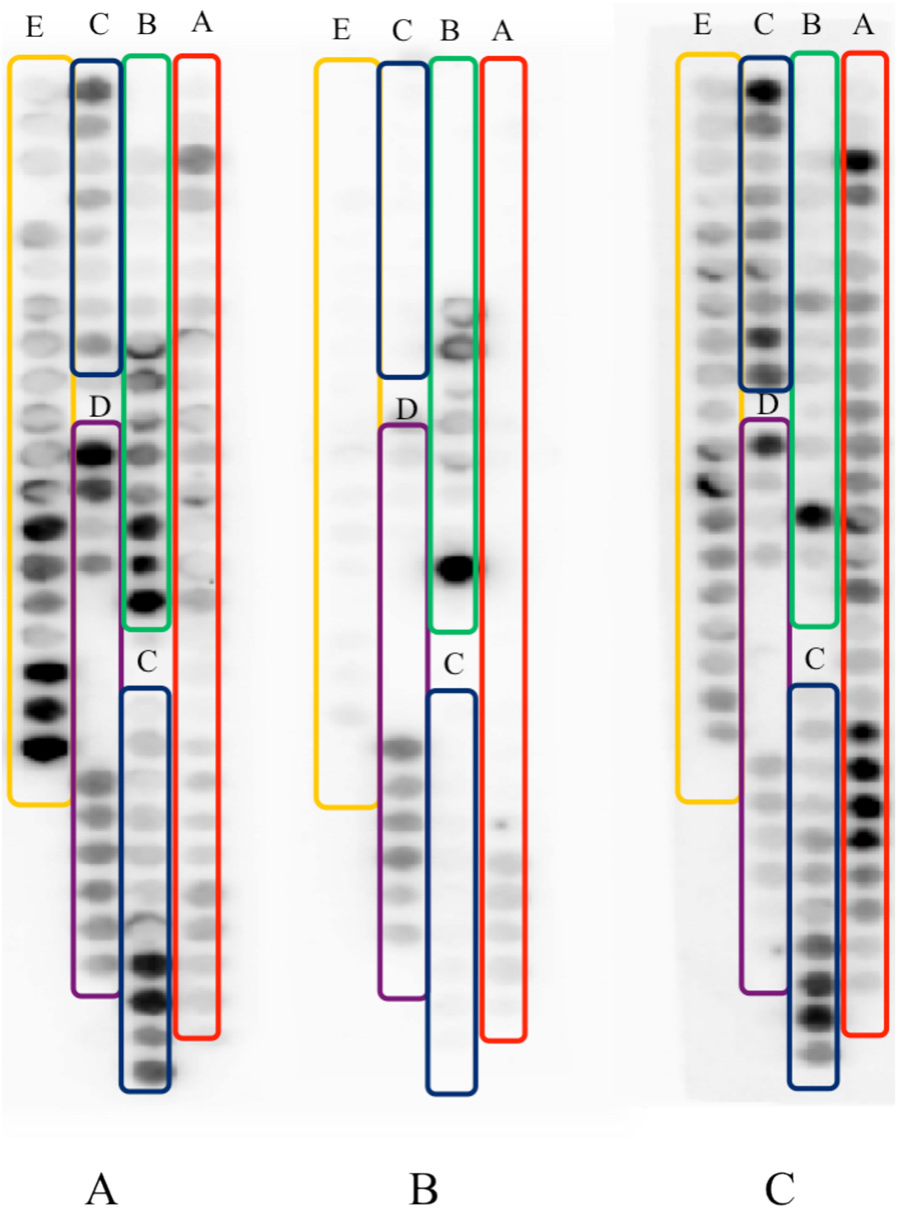
**Validated peptide candidates that may block TDP-43 binding determined using a high density peptide array.** The peptide mixture inhibits the binding between TDP-43 protein and the membrane. Membranes were incubated with **A**, TDP-43 protein; **B**, TDP-43 protein and a mixture of the five candidate peptides; **C**, TDP-43 protein and the scrambled peptide TC. The data show that the five candidate peptides can effectively inhibit the binding of TDP-43 to the TDP-43 membrane. The experiment was repeated three times with similar results.

To verify whether the five regions of TDP-43 identified through the array assay are actually involved in self binding of the protein, the five peptides indicated in Figure 1 were synthesized and tested for their ability to inhibit the binding between full length TDP-43 and the TDP-43 peptide array (Figure 2). A scrambled peptide TC was designed as a control. Peptide TC has the same length, net charge, and number of hydrophilic and hydrophobic amino acids as Peptide C. It has a random order and substitutions with the structure disrupting amino acids of original peptide. The results showed that a mixture of the five peptides blocked the interaction between TDP-43 and its peptide array (decrease by 76.05% ± 3.46% compared to control) (Figure 2A, B). However, the control peptide TC was not able to block the interaction (Figure 2C).

### Synthetic peptides inhibit aggregation of overexpressed mutated TDP-43 in HeLa cells

**Figure 3:**
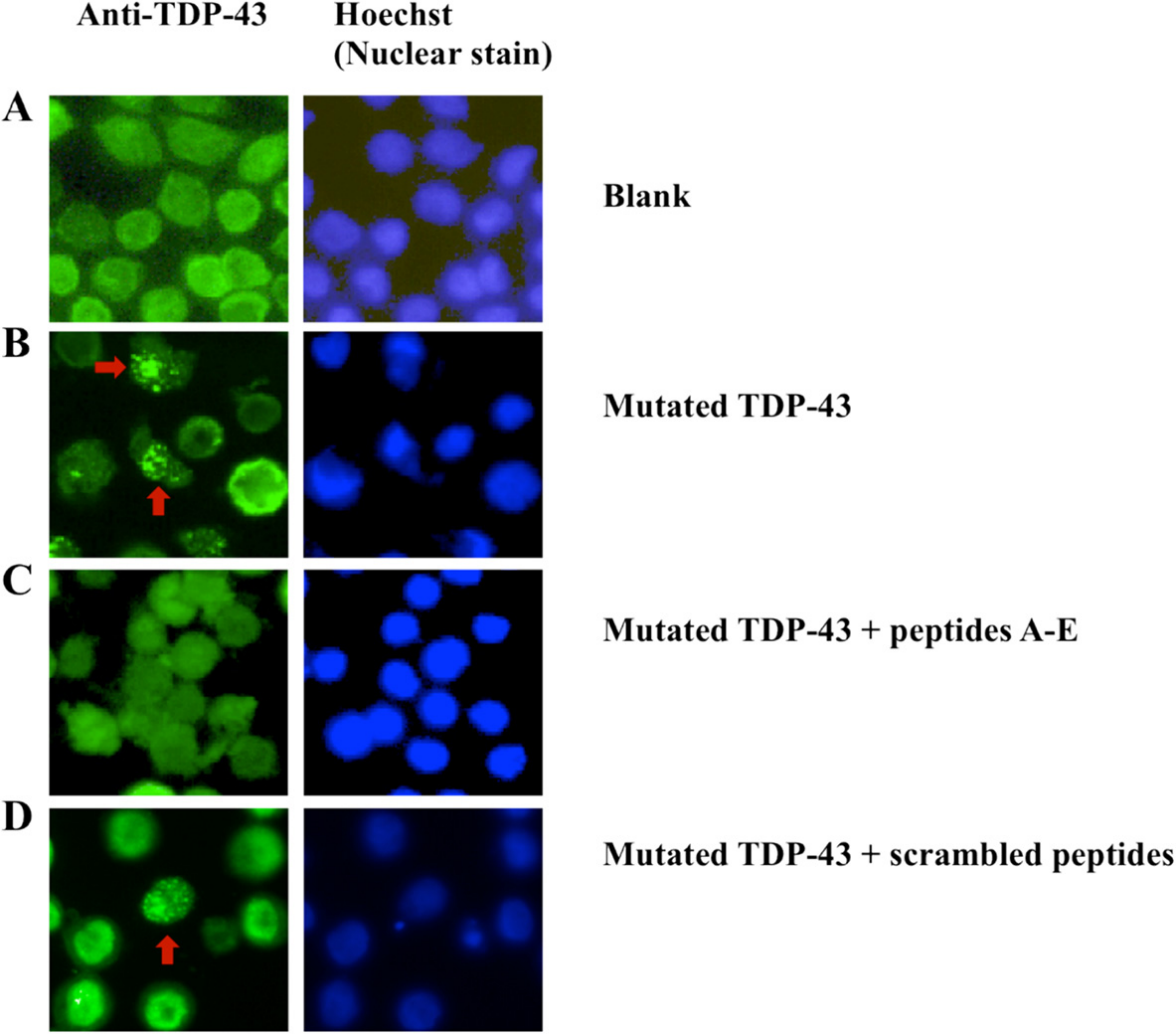
**Expression of mutated TDP-43 (deletion of 187–192 AA) results in the formation of inclusions.** Immunofluorescent detection of TDP-43 in HeLa cells with anti-TDP-43 antibody (left panel, green), nuclear staining by Hoechst (right panel, blue). **A**: untransfected HeLa cells; **B**: HeLa cells examined 48 h post transfection with mutated TDP-43; **C** and **D**: HeLa cells examined 48 h post transfection with mutated TDP-43, followed by addition of the mixture of five peptides (10 μM) **(C)** or the scrambled peptide TC (10 μM) **(D)** to the culture medium. The peptides effectively inhibited aggregation of TDP-43 in HeLa cells. The experiment was repeated three times with similar results.

To examine whether the peptides blocking interaction of recombinant TDP-43 protein with the peptide array could also inhibit TDP-43 aggregation in cells, a construct expressing a mutant TDP-43 lacking residues 187–192 [18] was transfected into HeLa cells. A construct expressing wild-type TDP-43 was used as a control. Immunohistochemical analysis was performed 48 hours after transfection. As shown in Figure 3, while all cells express wild-type TDP-43, only cells transfected with mutant TDP-43 showed TDP-43 aggregates (18.40% ± 0.62%), demonstrated by punctate staining in the nuclei.

**Figure 4:**
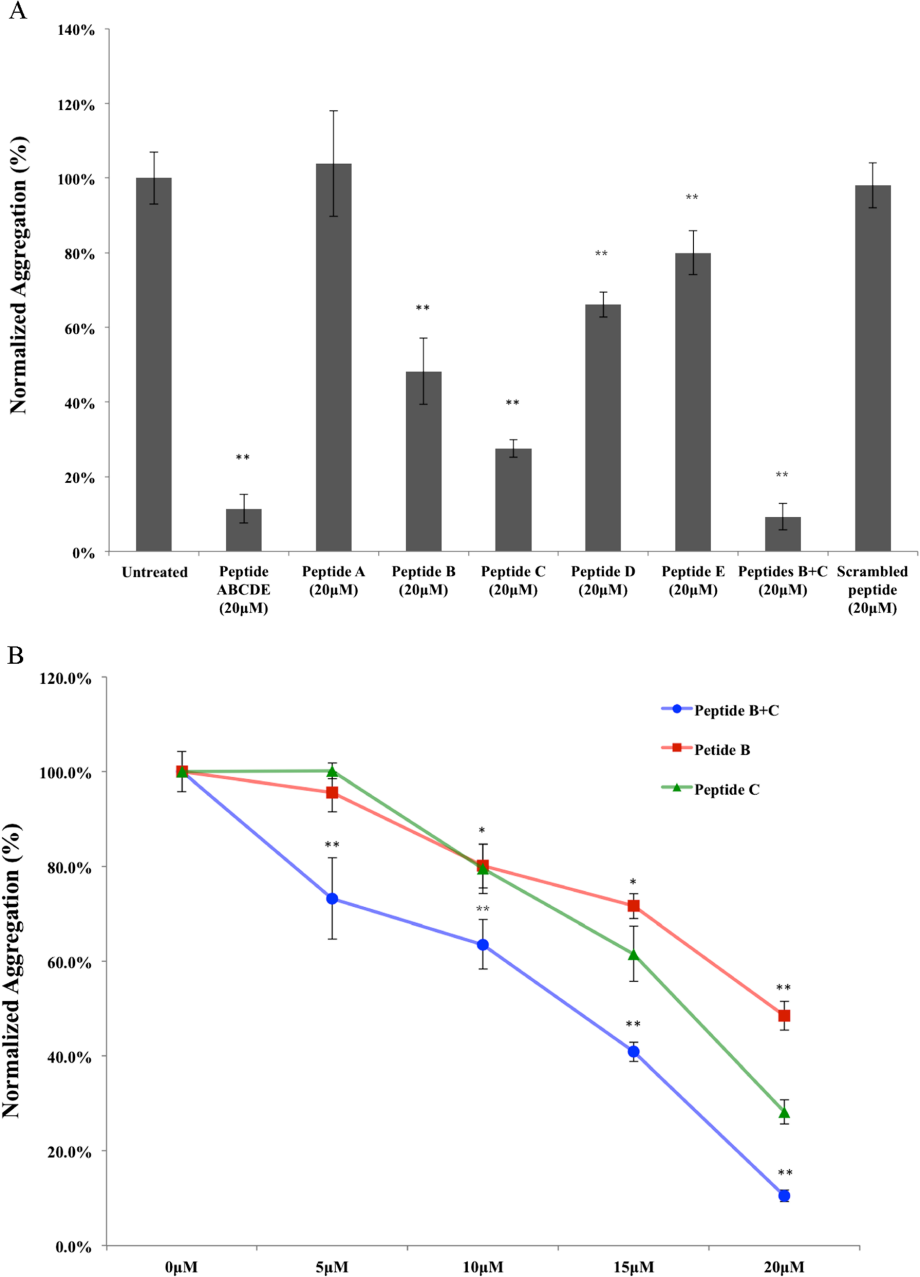
**Synthetic peptides can inhibit TDP-43 aggregation. A**: Peptide B, C and B + C combined were able to inhibit aggregation more efficiently than the other three peptide candidates. **B**: Peptide B, C and the B + C mixture inhibit aggregation in a concentration-dependent manner (*, p < 0.05; **, p < 0.01, one-way ANOVA). The data shown are from an experiment with three replicates.

To investigate whether the synthetic peptides could inhibit the aggregation of mutant TDP-43, N-terminally TAT conjugated peptides A, B, C, D, E and/or TC were added to the cell culture medium after transfection. The peptides were added either individually or combined together in various ways (peptide A-E mixture, or peptide B and C mixture). The TAT peptide has been used to deliver large molecules and small particles across both the plasma membrane and the nuclear membrane [24]. The number of mutant TDP-43 inclusions decreased in cells transduced with the peptides (Figures 3 and 4). Peptides B and C were found to be much more effective at reducing aggregation than the other synthetic peptides derived from TDP-43 or than the control TC peptide. Compared to untreated cells, cells with TDP-43 aggregates were reduced by 52% ± 8.85% (p < 0.01), 72% ± 2.33% (p < 0.01), and 91% ± 3.56% (p < 0.01) after treatment with Peptide B (20 μM), Peptide C (20 μM) and a Peptide B and C mixture (20 μM), respectively (Figure 4A). In addition, the peptides blocked aggregation in a concentration-dependent manner (Figure 4B). Peptides A, D, E or the scrambled control TC did not show statistically significant effects (Figure 4A). Peptides A-E applied to the untransfected cells had no effect on cell viability.

### Synthetic peptides inhibit aggregation of endogenous TDP-43 induced by arsenite stress in HeLa cells

**Figure 5:**
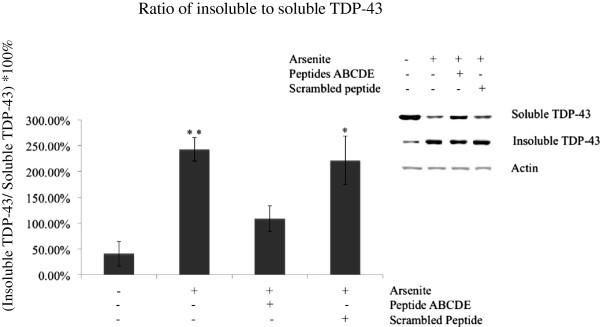
**Synthetic peptides decreased arsenite induced TDP-43 aggregation.** Insoluble and soluble TDP-43 levels were quantified under different conditions (basal, arsenite, arsenite + Peptide ABCDE, arsenite + Scrambled peptide) (*, p < 0.05; **, p < 0.01, one-way ANOVA). The data shown are from an experiment with three replicates.

To investigate whether our peptides could inhibit aggregation of wild-type TDP-43 under stress conditions, HeLa cells were treated with arsenite with or without addition of synthetic peptides. After exposure to 0.5 μM arsenite with or without 10 μM of the synthetic peptide mixture (ABCDE) for 4 hours, the cell lysates were sequentially extracted and separated into soluble and insoluble proteins. The relative ratio of insoluble to soluble TDP-43 was used as a measure of TDP-43 aggregation propensity. After the treatment with arsenite, soluble endogenous TDP-43 was transformed into predominantly insoluble protein and showed a significant increased aggregation propensity (243.23 ± 22.97%) compared with control cells without treatment (41.02 ± 24.02%) (Figure 5). When the synthetic peptides (Peptide A-E mixture) were coincubated along with the arsenite, the ratio of insoluble to soluble TDP-43 decreased to 109.13 ± 25.00%. Treatment with the scrambled peptide did not lead to any reduction compared to non-treated arsenite-stressed cells (221.57% ± 46.76%) (Figure 5). These results show that our synthetic peptides can reduce the formation of insoluble wild-type TDP-43 under stress conditions.

### The inhibition of aggregation cannot prevent cell death

**Figure 6:**
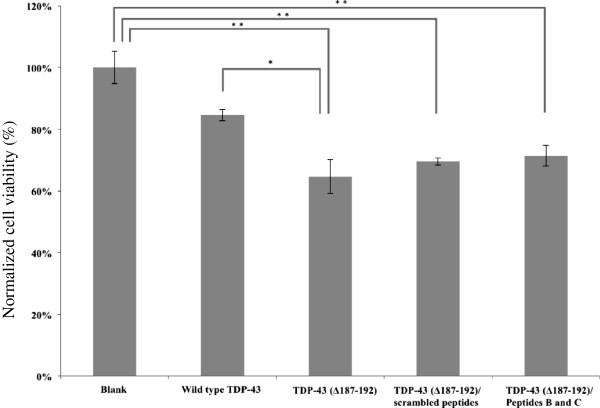
**Assessment of cell viability using the MTT assay.** Cells were transfected with either wild type or mutated TDP-43 plasmids. Peptides B and C were unable to prevent cell death. Cell viability was normalized to the basal condition (*, p < 0.05; **, p < 0.01, one-way ANOVA). The data shown are from an experiment with three replicates.

To ask whether TDP-43 aggregation is the cause of cell death, we used MTT and TUNEL assays to examine cell viability in cells expressing the mutant TDP-43. Overexpression of the mutant TDP-43 did indeed induce significant cell death (64.60 ± 5.44% cell viability, p < 0.01) compared to untreated cells. Treatment with Peptide B and C, which significantly reduced the aggregation, led to similar levels of cell viability (71.37% ± 3.30%, p < 0.01) as was found in untreated and scrambled peptide treated cells (68.90% ± 2.96%, p < 0.01) (Figure 6).

**Figure 7:**
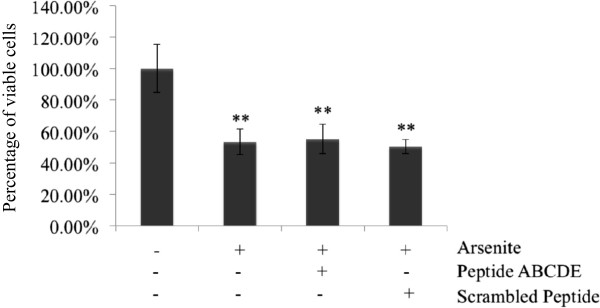
**Synthetic peptides did not rescue arsenite induced cell toxicity.** Cell viability was assessed using the MTT assay and normalized to the basal condition (*, p < 0.05; **, p < 0.01, one-way ANOVA). The data shown are from an experiment with three replicates.

Similar results were observed in the cells in which wild-type TDP-43 aggregation was reduced after treatment with arsenite and synthetic peptides. Treatment with arsenite induced significant HeLa cell death (53.32 ± 8.13% cell viability, p < 0.01) compared to the basal condition. Our synthetic peptides decreased insoluble TDP-43 but did not prevent cell death (55.10 ± 9.38% cell viability, p < 0.01) (Figure 7). These results indicate that TDP-43 aggregation was associated with cell death, but inhibition of TDP-43 aggregation could not rescue or prevent cell death.

**Figure 8:**
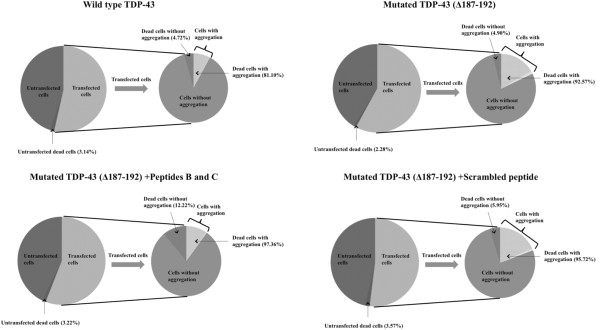
**Schematic flowchart of the cell death studies.** Cell death was identified using the TUNEL assay in HeLa cells. Cells were transfected with GFP and the wild type TDP-43 or mutated TDP-43 plasmid. About 1000 cells of each sample were counted. After treatment with Peptide B and C, the amount of cells with aggregation decreased. However, despite the reduction in aggregation, increased cell death was observed in the non-aggregated cells. This suggests that the peptides can inhibit aggregation but not cell death. In addition, the very low percentage of cell death in untransfected cells suggests the peptides are not toxic to cells.

To further elucidate the relationship between the cell death and TDP-43 aggregation, the cells were triple-labeled with TDP-43, GFP and the cell death marker, TUNEL. The numbers of transfected cells, aggregation-positive cells, or TUNEL-positive cells were counted by a blinded independent investigator. We measured four fields and 1000–1200 cells per microscope slide. We found that almost all the cells with TDP-43 aggregation that were untreated, Peptide B and C treated, or scrambled peptide (TC) treated showed TUNEL positive signals. In addition, even though Peptides B and C combined reduced TDP-43 aggregation, a much higher percentage of dead cells were observed in Peptides B and C treated cells without aggregation (12.22% ± 0.75%) than in scrambled peptide treated cells (5.95% ± 0.95%). Finally, we also measured cell death in cells without mutant TDP-43 overexpression but treated with the peptides. Cell death percentage in the untransfected cells of wild-type TDP-43, mutant TDP-43, mutant TDP-43 plus peptides BC and mutant TDP-43 plus scrambled peptide groups were 3.14% ± 0.56%, 2.28% ± 0.36%, 3.22% ± 0.35%, and 3.57% ± 0.39%, respectively (Figure 8). There was no difference among all of these groups, indicating that the peptides themselves were noncytotoxic.

## Discussion

Here we report the development of synthetic peptides that can reduce the aggregation of mutant TDP-43 and can prevent the formation of stress induced wild type TDP-43 inclusions in HeLa cell models. We were concerned about “off-target” interactions of our peptides. We found no other proteins sharing the same sequences of TDP-43 to which peptides bind after searching the NCBI molecular biology database. We therefore think that off-target interactions may be less likely.

Our findings confirm an earlier report by Nonaka and colleagues in SY5Y cells by showing that mutated TDP-43 (∆187-192) can induce TDP-43 aggregation and cell death in HeLa cells [18]. However, although many TDP-43 mutations have been found, the majority of ALS and FTLD-U cases have no known TDP-43 mutations and instead, it is wild-type TDP-43 that aggregates in these conditions [25]. Accordingly, we tested our peptides on a second model in HeLa cells in which the formation of insoluble endogenous TDP-43 aggregates was induced by oxidative stressor arsenite [26]. Increased TDP-43 aggregation was observed and quantified using immunocytochemical and biochemical methods (separation of soluble and insoluble protein). Importantly, while the peptides reduced both mutant TDP-43 and endogenous TDP-43 aggregation induced by stress in a concentration-dependent manner, they did not prevent or reduce the cytotoxicity caused by overexpression of the mutant TDP-43. These data suggest that while TDP-43 aggregation is associated with the process of cytotoxicity, it is not the cause of cell death in our model.

Our results indicating that aggregation may not be the causative factor for cytotoxicity in cells expressing mutant TDP-43, appear consistent with other emerging evidence that aggregation is not necessarily the cause of cellular toxicity. Previous work from our lab [27] showed that knockdown of progranulin in mouse cortical neurons induced TDP-43 translocation from nucleus to the cytoplasm and resulted in enhanced vulnerability to several stressors, including H_2_O_2_ and NMDA. Despite this increased neuronal vulnerability, we did not observe either nuclear or cytoplasmic TDP-43 aggregation. These results suggesting that reduced levels of TDP-43 in the nucleus may be critical for cytoxicity are consistent with evidence reviewed by Z.S. Xu in a recent paper [28]. These results are also consistent with those of Barmada et al. who recently established a TDP-43 proteinopathy model by transfecting a mutant form of TDP-43 in rat primary cortical neurons. They observed that their mutant TDP-43 is translocated from nucleus to cytoplasm and observed increased cell death. However, they found no aggregation or inclusions in their neurons [29]. Recent evidence from transgenic mice has also been reported and appears consistent with this idea. Expression of a mutant TDP-43 plasmid induced neuron death and degeneration, but did not lead to cytoplasmic inclusions [30]. Moreover, some studies with other proteins have shown that aggregation may even serve a protective role. In Huntington’s disease, for instance, mutant Huntington protein (mHtt) is toxic when it is soluble [31,32]. Overexpression of mHtt in HEK293 cells caused aggregation but had no effect on cell survival. However, when mHtt was overexpressed with the small guanine nucleotide-binding protein Rhes, it showed decreased aggregation but increased cell death [33]. Together those results are leading to the view that protein aggregation is not an obligatory factor leading to cytotoxicity.

Protein aggregation has been observed in a number of neurodegenerative diseases. While aggregation is generally to be associated with cytotoxicity, it can also be a phenomenon associated with excessive ER stress, which is caused by an unusually large amount of misfolded proteins in the cell [34-38]. In fact, it is now clear that overexpression of wild-type TDP-43 can also cause aggregation and cytotoxicity as observed by us (Figure 6) and others [16,39]. Expression of large amounts of wild-type TDP-43 might cause an ER stress response and then initiate cell death. By reducing the protein-protein interaction that leads to aggregation, the present study, to our best knowledge, is the first attempt to separate the phenotype of TDP-43 aggregation from that of cytotoxicity. It also provides insights into the mechanism of TDP-43 toxicity in FTD and ALS. Our study suffers from the limitation that our experiments were performed in a HeLa cell model. FTLD and ALS are diseases of neurons, and moreover of specific populations of neurons. Our experiments need to be extended and replicated in selected populations of cultured neurons. In addition, further studies in transgenic mice will be needed to determine whether our peptides can also reduce TDP-43 aggregation *in vivo*. Furthermore, the same approach as the one taken here may be used to study the role of aggregation of other proteins, e.g. Huntingtin, α-synuclein, β-amyloid, in other neurodegenerative diseases.

## Conclusion

In this study, we are the first group to identify the regions in TDP-43 protein that involve in its self-aggregation. Peptides derived from those regions can effectively reduce the aggregation in cells transfected with an aggregation prone mutant TDP-43 or treated with arsenite. We then showed that reducing TDP-43 aggregation did not protect cells from cell death caused by either the expression of mutant TDP-43 or arsentie treatment. Thus, our study is the first to separate aggregation of TDP-43 from other factors involved in TDP-43 proteinopathy and demonstrate that TPD-43 aggregation may not be the cause of cytotoxicity.

## Methods

### Protein array

The peptides were synthesized on derivatized cellulose-based membranes (Intavis AG, Köln, Germany) by the UBC Peptide Synthesis facility using a previously described protocol [40]. The peptide scans were performed by synthesizing overlapping 12 or 14-mer peptides spanning the entire 414 amino acid sequence of TDP-43 with a frame shift of 2 amino acids per spot. The TDP-43 protein was purified from E. coil overexpressing the human TDP-43 gene. Membranes were blocked with 5% sucrose and 4% nonfat dry milk in Tris-buffered Saline Tween-20 (TBST) for 4 h and then incubated with TDP-43 protein (3–10 μg/ml) or peptides overnight at 4°C. Then membranes were incubated with rabbit polyclonal TDP-43 antibody (1:5000; Protein Tech Group, Chicago, IL) overnight at 4°C. After washing three times for 15 min, membranes were incubated with donkey anti-goat conjugated with donkey anti-rabbit IgG conjugated with horseradish peroxidase (1: 5000; R&D Systems, Minneapolis, MN) for 3 h at room temperature. Membranes were then washed three times for 15 min and protein interaction was visualized with an enhanced chemiluminescence reaction assay (PerkinElmer Life Sciences).

### Cell-penetrating peptide synthesis

Cell-penetrating peptides consisting of the truncated TAT domain at the N-terminal were synthesized by GL biochem Ltd. (Shanghai) and purified by HPLC. TAT, the trans-acting activator of transcription of the human immunodeficiency virus (HIV-1), has been used as an efficient way of delivering proteins or peptides into cells [41]. The resulting peptides were more than 90% pure and verified by mass spectrometry. All the peptides were first dissolved in DMSO and further diluted in water before usage.

### Blocking assay of TDP-43 interaction with the membrane

Membranes were incubated with the mixture of TDP-43 protein (5 μg/ml) and/or single synthetic peptides (100 μg/ml) overnight at 4°C. Then the membranes were incubated sequentially with primary antibody and secondary antibody and washed with TBST. The protein interaction was visualized using an enhanced chemiluminescence reaction assay (PerkinElmer Life Sciences). Optical densities of each peptide array were measured using NIH ImageJ software.

### Cell cultures, transfection and treatment

HeLa cells were obtained from the American Type Culture Collection (ATCC) and grown in Dulbecco’s modified Eagle’s medium (DMEM; Sigma Chemical Co., St. Louis, MO) supplemented with 10% fetal bovine serum (Gibco-BRL, Grand Island, NY) and 1% antibiotics (Gibco-BRL, Grand Island, NY). Cultures were maintained at 37°C in a humidified incubator (NuAir, Plymouth, MN) with 5% CO_2_. Wild type TDP-43 plasmid and mutant TDP-43 plasmid lacking residues 187–192 were obtained from Dr. Nonaka and Dr. Arai. The plasmids were transfected into the HeLa cells using the Lipofectamine 2000 system (Invitrogen, Carlsbad, CA) according to the manufacturer’s instructions. After incubating the cells with the transfection mixture for 5 hours, the medium was replaced with fresh DMEM (10% fetal bovine serum) with 2.5–20 μM synthetic peptides. After 24 hours, the medium was replaced with fresh peptides. The cells were returned to the incubator for an additional 24 hours before study.

For studies using arsenite, arsenite (Fisher scientific CO., Pittsburg, PA) was added to the cell culture medium to a final concentration of 0.5 μM with or without the presence of synthetic peptides. The cells were then returned to the incubator for 4 hours before harvesting and analysis.

### Immunofluorescence studies

Cells were fixed with pre-warmed 4% Paraformaldehyde (PFA; Sigma, Saint Louis, MO) containing 4% sucrose (Sigma, Saint Louis, MO) in PBS for 10 minutes at room temperature and permeabilized with 0.1% Trition X-100 (Sigma, Saint Louis, MO) in PBS for 2–3 minutes at room temperature. Then the reaction was blocked with 10% Bovine serum albumin (BSA; Invitrogen, Carlsbad, CA) in PBS for 1 hour at room temperature and incubated with a primary antibody against TDP-43 (1:150; ProteinTech, Chicago, IL) overnight at 4°C. Reactions were visualized with anti-rabbit antibody conjugated with Alexa 488 (Invitrogen, Carlsbad, CA). Cell nuclei were labeled with DAPI (1:10000; Invitrogen, Carlsbad, CA) for 2–3 minutes at room temperature. Images were obtained with an Olympus Fluoview FV1000 Confocal scanning microscope.

### MTT assay for cell viability

The viability of cultured cells was assessed using the MTT assay. MTT (3-(4,5-dimethylthiazol-yl)-2,5-diphenyltetrazolium bromide) (Sigma, Saint Louis, MO) was added to each well (20 μl, 5 mg/ml). After 4 h incubation, cells were lysed overnight. The optical density of test and control samples was measured at 590 nm absorbance with a reference filter of 750 nm using a “uQuant” microplate spectrophotometer (Bio-Tek Instruments, USA). The absorbance was translated into cell viability ratios for comparison: cell viability ratio = (test sample absorbance/control sample absorbance) × 100%.

### TUNEL assay for cell viability

Deoxynucleotidyl-transferase-mediated dUTP nick end-labeling (TUNEL) staining was performed using an *in situ* cell death detection kit (Roche Applied Science, IN). Cells were seeded on poly-D-lysine coated cover slips at a density of 2.5 × 10^4^ cells per well in 24 well plate. After treatment, cells were fixed and stained with anti-TDP-43 (1:150; ProteinTech, Chicago, IL), anti-GFP (1:1000; Invitrogen, Carlsbad, CA), Alexa 647 anti-rabbit (1:1000; Invitrogen, Carlsbad, CA) and Alexa 488 anti-chicken (1:1000; Invitrogen, Carlsbad, CA). The enzyme solution (terminal transferase) and label solution were mixed in a volume ratio of 1:9 to obtain the TUNEL reaction mixture. The cells were incubated with the 50 μl TUNEL reaction mixture at 37°C for 1 hour and then washed three times with PBS. Then the cover slips were mounted on glass slides with antifade reagent (Invitrogen, Carlsbad, CA). Images were obtained with an Olympus Fluoview FV1000 Confocal scanning microscope.

### Biochemical fractionation

To examine the effect of arsenite on solubility of endogenous TDP-43, preparation of soluble/insoluble protein were performed. HeLa cells were washed twice with cold PBS, lysed in cold lysis buffer (PBS with 1% Triton X-100, 10 μg/ml aprotinin, 0.5 mM PMSF). Cell lysates were rotated for 15 min and then centrifuged at 55,000 rpm for 15 min at 4°C. The supernatants were collected as soluble proteins. To prevent contamination caused by carrying over, the pellets were re-centrifuged at 55,000 rpm for 15 min at 4°C and washed once with lysis buffer. Then the pellets were lysed in PBS with 1% SDS and sonicated. The supernatants were collected as insoluble protein. Soluble and insoluble proteins were analyzed by immunoblotting.

### Immunoblotting

Protein concentrations of cell lysate and medium were measured using a standard BCA assay (Bio-Rad) as per the manufacturer’s instructions. Proteins were separated in SDS–PAGE (10%) and transferred to nitrocellulose membranes (Bio-Rad) at 4°C. Membranes were blocked with 5% nonfat dry milk in TBST for 1 hour, then incubated in 5% nonfat dry milk/TBST overnight at 4°C with antibodies for TDP-43 or β-actin (rabbit IgG, 1:1000; Cell Signaling, Danvers, MA). After washing with TBST, membranes were incubated with anti-rabbit secondary antibody conjugated with horseradish peroxidase for 1 hour at room temperature. Blots were developed using an enhanced chemiluminescence reaction assay.

### Statistical analysis

All data were expressed as mean ± SD and analyzed by one-way ANOVA. A value of p < 0.05 was considered statistically significant.

## Competing interests

The authors declare that they have no competing interests.

## Authors’ contributions

RL, WJ and MSC conceived and designed the experiments. RL performed the experiments and analyzed the data. GY provided assistance in application of immunofluorescence methods. TN and TA were involved in plasmid construction. RL, WJ and MSC drafted and edited the manuscript. All Authors read and approved the final manuscript.
